# Passive Sampling Tool for Actinides in Spent Nuclear Fuel Pools

**DOI:** 10.1021/acsomega.2c01884

**Published:** 2022-06-02

**Authors:** Joshua D. Chaplin, Marcus Christl, Marietta Straub, François Bochud, Pascal Froidevaux

**Affiliations:** † Institute of Radiation Physics, Lausanne University Hospital and University of Lausanne, 1 Rue du Grand-Pré, Lausanne CH-1007, Switzerland; ‡ Laboratory of Ion Beam Physics, ETH Zürich, Otto-Stern-Weg 5, Hönggerberg, Zürich 8093, Switzerland

## Abstract

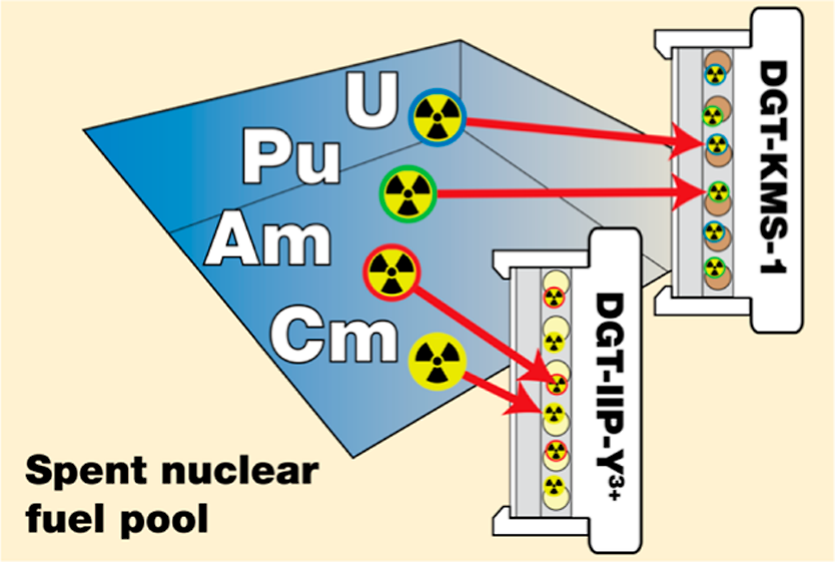

Spent nuclear fuel must be carefully managed to prevent pollution of the environment with radionuclides. Within the framework of correct radioactive waste management, spent fuel rods are stored in cooling pools to allow short-lived fission products to decay. If fuel rods leak, they liberate radionuclides into the cooling water; therefore, it is essential to determine radionuclide concentrations in the pool water for monitoring purposes and to plan the decommissioning process. In this work, we present, to our knowledge, the first passive sampling technique for measures of actinides in spent nuclear fuel pools, based on recently developed diffusive gradients in thin-film (DGT) configurations. These samplers eliminate the need to retrieve and handle large samples of fuel pool water for radiochemical processing by immobilizing their targeted radionuclides in situ on the solid phase within the sampler. This is additionally the first application of the DGT technique for Cm measure. Herein, we make the calibrated effective diffusion coefficients of U, Pu, Am, and Cm in borated spent fuel pool water available. We tested these samplers in the fuel pool of a nuclear facility and measured samples using accelerator mass spectrometry to provide high-precision isotopic reports, allowing for the first independent implementation of a recently developed technique for dating nuclear fuel based on its Cm isotope signature.

## Introduction

Determining the radionuclide content in the water of spent fuel pools (SFPs) at nuclear facilities is important in case a fuel rod may be leaking and liberating radionuclides. Radionuclide levels present in the SFP have implications for fuel reprocessing and planning the decommissioning process. During decommissioning, it would be useful to distinguish the dissolved labile fraction of radionuclides in the SFP water from the particle-immobilized fraction to assess the effectiveness of adsorbents used to capture radionuclides from the water. Additionally, activities of β/γ-emitting radionuclides in the SFP water can be orders of magnitude above those of α-emitters. Therefore, retrieving enough SFP water for *ex situ* laboratory analysis of α-emitting actinides can complicate handling of the sample. A more selective sampling solution for the actinides could therefore ease this task.

We present an alternative *in situ* sampling solution for measuring the labile fraction of the main actinides of interest (U, Pu, Am, and Cm) in SFP water based on the diffusive gradients in the thin-film (DGT) technique. These samplers eliminate the need for the retrieval of SFP water samples, while they calculate a time-weighted average concentration (*c*_DGT_) for their target actinides. Selectively immobilizing the target actinides from the SFP water onto the solid phase within the sampler significantly eases sample handling.

Herein, we present a technical note which calibrates and evaluates the recently developed KMS-1 and IIP-Y^3+^ DGT configurations^1^ for use in borated SFP waters. In this work, we make the diffusion coefficients (*D*) available for the major actinides (U, Pu, Am, and Cm) in simulated SFP water based on diluted boric acid (H_3_BO_3_). We also extend the scope of the DGT technique to measure Cm using the IIP-Y^3+^ DGT configuration.^1^ This is important for SFP measures as Cm isotopes are significant α-emitting sources present in spent fuel.

We deployed KMS-1 and IIP-Y^3+^ DGT samplers in the SFP of a nuclear facility and measured the actinides in the DGT resin-gel eluates by ultrasensitive accelerator mass spectrometry (AMS). AMS allowed us to produce a comprehensive isotopic report, including for the spectrum of Cm isotopes ^242–246^Cm following a short deployment period in the SFP of 24 h. This is not possible using α-spectrometry due to the overlapping α-emission energies of ^243^Cm and ^244^Cm, and ^245^Cm and ^246^Cm. Employing AMS also allowed us to assess the validity of a recently developed Cm dating method for nuclear fuel.^2^ We also report a novel radiochemical methodology to measure Am and Cm isotopes in the same fraction by AMS herein, enabling the production of *c*_DGT_ for isotopes of both elements with a single ^243^Am spike.

## Experimental Section

### Materials and Solutions

All reagents used were of analytical grade, from Sigma-Aldrich, Merck, or DGT Research (Lancaster, UK). The KMS-1 and IIP-Y^3+^ DGT sampling configurations were synthesized as described by Chaplin et al.^1^ Diffusive polyacrylamide gels, membrane filters, and the cross-linker solution were purchased from DGT Research. Boric acid stock solution (BASS), based on 4000 ppm B (pH 4.5), was prepared according to the composition of SFP at a nuclear facility with whom we collaborated for the practical deployments of samplers. BASS was used for laboratory validation experiments using a diffusion cell and fully assembled DGT samplers (see the following sections). Actinide standards (^233^U, ^238^Pu, and ^241^Am) and tracers (^232^U, ^242^Pu, and ^243^Am) were prepared by the Radiometrology Unit at the Lausanne University Hospital’s Institute of Radiation Physics and are traceable to NIST sources. ^243+244^Cm was retrieved from the eluate of a DGT sampler deployed in SFP water at the nuclear facility and used as the analyte in a diffusion cell experiment.

### Laboratory Validation: Diffusion Cell Experiments

A custom-built diffusion cell was used to determine the diffusion coefficient (*D*), following the procedure described by Cusnir et al.^3^ Samples taken from the A and B sections of the diffusion cell were evaporated to dryness and prepared for sequential radiochemical extraction of the Pu fraction, then the U fraction, and then a combined Am + Cm fraction according to the following “U and Pu Radiochemistry” and “Am and Cm Radiochemistry” sections.

### Practical Deployments in SFP

Custom 105 cm^2^ surface-area DGT-sampler housings as described by Cusnir et al.^4^ were deployed in the SFP of a nuclear facility for 24 h. The samplers were held within a custom-built pure stainless-steel support to prevent reaction with the SFP water. Samplers were retrieved, washed thoroughly with deionized water, and sealed in plastic bags. The radiation dose (μSv·hr^–1^) on contact with the plastic bag was recorded to determine if the samplers had also captured significant quantities of β- and γ-emitting fission and/or activation products along with the actinides. This step is critical to inform the appropriate handling and transport of the package.

Upon receipt, 105 cm^2^ surface-area DGT samplers were unpacked and checked for the absence of surface contamination using a surface contamination monitor for β/γ and a wipe test. Once confirmed as safe to handle, samplers were disassembled, and the resin gels were retrieved. U and Pu were eluted from the KMS-1 resin gels by immersion overnight in 50 mL of 8 M HNO_3_ with five pipette drops of H_2_O_2_. Am and Cm were eluted from IIP-Y^3+^ resin gels by immersion overnight in 3 M HCl.

Aliquots of the eluates were prepared for γ- and α-spectrometry to estimate the maximum activity present. This is critical to ensure that the AMS instrument is not contaminated by introducing a high-activity sample into its ion source. Based on these measures, suitable aliquots of the resin-gel eluates were manufactured into AMS targets to measure individual isotopes that have overlapping α-emission energies and cannot be separately distinguished using α-spectrometry (^240^Pu, ^239^Pu, ^233^Cm, ^244^Cm, ^245^Cm, and ^246^Cm) and/or low-abundance and long-lived isotopes that require ultrasensitive mass spectrometric measure (^236^U, ^245^Cm, and ^246^Cm)

### U and Pu Radiochemistry

Pu and then U were sequentially extracted from samples according to the procedures described in detail by Chaplin et al.^1^ In brief, Pu in the sample was fixed to Pu(IV) in 8 M HNO_3_ and extracted on Bio-Rad AG 1-X4 resin. U was separated from the column waste in 1 mL pipette cartridge microcolumns packed with UTEVA resin. The eluate residues from the columns of diffusion cell experiments were prepared for measure by α-spectrometry (see the “Calculation of D: Laboratory Validations” section). Column eluate residues from samplers deployed in the SFP at the nuclear facility were prepared for measure by AMS (see the “AMS Target Manufacture” section).

### Am and Cm Radiochemistry

Am and Cm were eluted in the same radiochemical fraction according to the procedure for Am elution from IIP-Y^3+^-packed cartridges reported by Chaplin et al.^1^ In brief, eluates from laboratory validation experiments were traced with ^243^Am for measure by α-spectrometry and evaporated to dryness. Prior to radiochemical separation on IIP-Y^3+^ cartridges, eluates of the IIP-Y^3+^ resin gels from DGT samplers deployed in the SFP at the nuclear facility were divided into two separate aliquots. The spiked aliquot was traced with ^243^Am for measure by AMS, while the unspiked aliquot was not traced. Both fractions were evaporated to dryness.

### Calculation of *D*: Laboratory Validations

The analytes for diffusion cell experiments (^233^U, ^238^Pu, ^241^Am, and ^243+244^Cm) were used at suitable activity concentrations for measure of samples by passivated implanted planar silicon α-spectrometers (Mirion Technologies). The eluates from radiochemical separations were evaporated to dryness and electrodeposited on stainless-steel discs according to the procedure reported by Bajo et al.^5^*D* from diffusion cell experiments was calculated using eq 1 (below), where  is the *D* calculated at the temperature of experimentation.  was then normalized to 25 °C using the Stokes–Einstein correction, as previously reported for these analytes according to eqs 2–4 of Chaplin et al.^1^
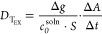
1

Here,  is the area of the diffusive window (cm^2^),  is the concentration of the analyte in solution (taken at the beginning of the experiment, mBq·mL^–1^), and slope  is the activity (, mBq) diffused into section B of the cell against time (, seconds).  is the material diffusion layer thickness (cm), comprising the filter membrane (0.014 cm), diffusive gel thickness (0.039 or 0.078 cm), and the diffusive boundary layer, .  was considered negligible for diffusion cell experiments due to thorough mixing of the solution.

*c*_DGT_ from sampler deployments was calculated according to eq 2
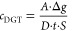
2

Here,  is the activity or mass of the analyte isotope in the resin-gel eluate;  for sampler deployments includes an additional of  0.049 cm for each actinide, as reported for Pu by Cusnir et al.^4^ and Am by Chaplin et al.;^1^*t* is the exposition time (s), and *S* is the surface area of the exposed filter membrane of DGT samplers.

### AMS Target Manufacture

Radiochemically purified U, Pu, and spiked and unspiked Am + Cm fractions from SFP sampler deployments were evaporated to dryness and the residue was resuspended in 20 mL of 1 M HCl. 1 mL of the Fe(III) stock solution (2 mg·mL^–1^) was added while mixing at 500 rpm. Concentrated (30%) NH_4_OH was added dropwise to coprecipitate the actinides with Fe-hydroxide. The precipitate was centrifuged, desiccated at 80 °C until dry, and baked at 650 °C for at least 4 h to convert Fe into the oxide form. 2–3 mg of Nb powder was added, and the sample was homogenized using a spattle. The powder was compressed into a modified NEC cathode according to ETH Zürich (ETHZ)’s Laboratory of Ion Beam Physics internal procedure.^6^ U and Pu measurements were normalized, respectively, to ETH Zürich’s in-house ZUTRI and CNA standards.^6^

AMS measurements were performed using ETHZ’s actinide-optimized TANDY facility.^6^ An increased stripper gas pressure was used to sufficiently suppress potential isobaric interferences on the actinide atomic mass units (AMUs, e.g., ^232^Th^12^C^3+^ on AMU 244), as previously reported for optimized AMS measure of Am and Cm.^7^ Each AMU was counted using cyclic repetition. For measures of the Am + Cm fraction, AMU 241 and AMU 243 were tuned using standards produced from certified NIST-traceable ^241^Am and ^243^Am sources provided by the Radiometrology group of Lausanne University Hospital’s Institute of Radiation Physics. For measures of the Am + Cm fractions, AMU 244 was additionally tuned using ETHZ’s ^244^Pu standard.

### Calculation of *c*_DGT_: Deployments in the Nuclear Facility SFP

*c*_DGT_ for analytes in the nuclear facility SFP water was calculated according to eq 2, using the  for the relevant element calibrated for boric acid from diffusion cell experiments (Table 1) and corrected to the temperature of the SFP during exposition.  for ^236^U and Pu isotopes (^239–241^Pu) were directly measured according to the relevant AMU in the U and Pu fractions.

**Table 1:** Diffusion Coefficients of the Actinides in BASS

analyte	***D*** (× 10^–5^ cm^2^.s^-1^)	*R*^2^ (diffusion)
^233^U	3.94 ± 0.19	0.9718
^238^Pu	1.39 ± 0.18	0.9743
^241^Am	3.36 ± 0.18	0.9906
^243+244^Cm	3.15 ± 0.29	0.9925

To calculate  for ^241^Am and Cm isotopes, the added ^243^Am tracer () in the spiked aliquot needed to be distinguished from the atoms of AMU 243 present in the sample . The range of AMU 241–246 in both the unspiked and spiked aliquots was measured by AMS using cyclic repetition on each AMU. The atoms of each AMU present in the sample are marked  or  for the spiked aliquot.  is the isotopic composition of the unspiked aliquot, whose AMU 241/243 and AMU 243/244 ratios are, respectively
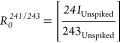
3
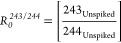
4

*R*_Spiked_ is the isotopic composition of the ^243^Am-spiked aliquot, whose AMU 241/243 or AMU 243/244 ratios are, respectively:
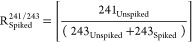
5
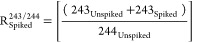
6

To distinguish the contribution of the ^243^Am tracer on the atoms of AMU 243 present in the unspiked aliquot , we can consider the relationship between  for AMU 241/243
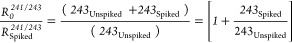
7243_Unspiked_ can therefore be derived from
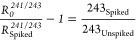
8

Giving
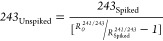
9

This allows  to be calculated for ^241^Am by giving  for ^241^Am as the atoms of AMU 241 present in the sample .
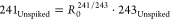
10

To allow  for ^244^Cm to be calculated by distinguishing the atoms of the ^243^Am tracer present in the sample, we can consider the relationship between  for AMU 243/244
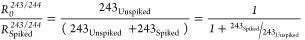
11

243_Unspiked_ can therefore be derived from
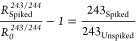
12

Giving
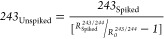
13

This allows  to be calculated for ^244^Cm by giving  for ^244^Cm as the atoms of AMU 244 present in the sample ().
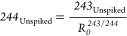
14

*c*_DGT_ values for ^242^Cm, ^243^Cm, ^245^Cm, and ^246^Cm were calculated from the ratio of the AMU of the isotope to *c*_DGT_ for ^244^Cm (using  as  for ^243^Cm). This method considers that all  in the sample is contributed from ^243^Cm and that any contribution from ^243^Am present in the SFP is negligible due to the short half-life of ^242^Am (*t*_1/2_ = 16 h), limiting production of ^243^Am during neutron irradiation. Additionally, a small known impurity of ^241^Am in our ^243^Am tracer was corrected for. This must be considered when employing this technique, in addition to any potential impurity of AMU 244 which may exist in the ^243^Am tracer used. We also note that this method considers that Am and Cm have the same ionization efficiency in the AMS ion source and that no correction is required for fractionation of the elements within the AMS beam. This can be reasonably assumed but remains uninvestigated.

### Application of the Cm Chronology Method

Using the ^244^Cm/^246^Cm (at/at) and ^245^Cm/^246^Cm (at/at) ratios of the Cm captured in the IIP-Y^3+^ DGT sampler, the age when the nuclear fuel was retired from its neutron flux was calculated according to a model reported by Christl et al.^2^ In brief, this is inferred from the decay of the shorter-lived isotope ^244^Cm (*t*_1/2_ = 18 a), which diminishes the ^244^Cm/^246^Cm ratio significantly over several years, while the ^245^Cm/^246^Cm ratio is essentially constant over several centuries as both isotopes are longer-lived (^245^Cm *t*_1/2_ = 8250 a, ^246^Cm *t*_1/2_ = 4723 a). A calibration curve has been fitted to a plot of log(^244^Cm/^246^Cm) versus log(^245^Cm/^246^Cm) for various types of nuclear fuel with a high linear correlation, regardless of the fuel type.^2^ Therefore, correcting the (^244^Cm/^246^Cm)/(^245^Cm/^246^Cm) of a sample of nuclear fuel of unknown age to this calibration curve will yield the age when it was retired from its neutron flux. This was performed by feeding the ^245^Cm/^246^Cm ratio of the sample (both spiked and unspiked aliquots were measured) into the power law relationship described by eq 15 to calculate the ^244^Cm/^246^Cm ratio at the origin [^244^Cm/^246^Cm]_0_
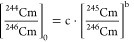
15

The Bateman equation (eq 16) was then used to calculate the time () in between  and the ^244^Cm/^246^Cm measured in the sample , indicating the point when the fuel was retired from its neutron flux:
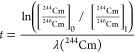
16

Here,  is the radioactive decay constant of ^244^Cm.

## Results and Discussion

### Determination of *D* Using the Diffusion Cell

Laboratory-determined *D* values for U, Pu, Am, and Cm in BASS at pH 4.5 are presented in Table 1, temperature corrected to 25 °C. *D* values for U, Pu, and Am are an order of magnitude higher than previously determined for each actinide in a buffered 10 mM NaNO_3_ solution and in seawater solutions.^1^ The additional mobility of the actinides in BASS compared to environmentally relevant solutions can be explained by the difference in the solution matrix, to some extent specifically given the much lower pH; the *D* values of U and Am are higher in acidic compared to alkaline solutions.^1^ Furthermore, there is no organic matter in SFP water which could change the speciation of the actinides; previous work has shown that the *D* values of Pu(IV) is reduced in the presence of humic acid.^8^ However, we consider that the much higher *D* for U, Pu, and Am/Cm in BASS is probably moreover due to the tendency for boric acid to form large clusters with strong H···(HO)_n_B hydrogen bonds, which will reduce the M^z+^-B(OH)_3_ interaction. Boric acid is also known to form complexes with either H-donor or anionic ligands, which in turn will increase the lability of the metal. This would make M^z+^ freely available for fast diffusion.

**Figure 1:**
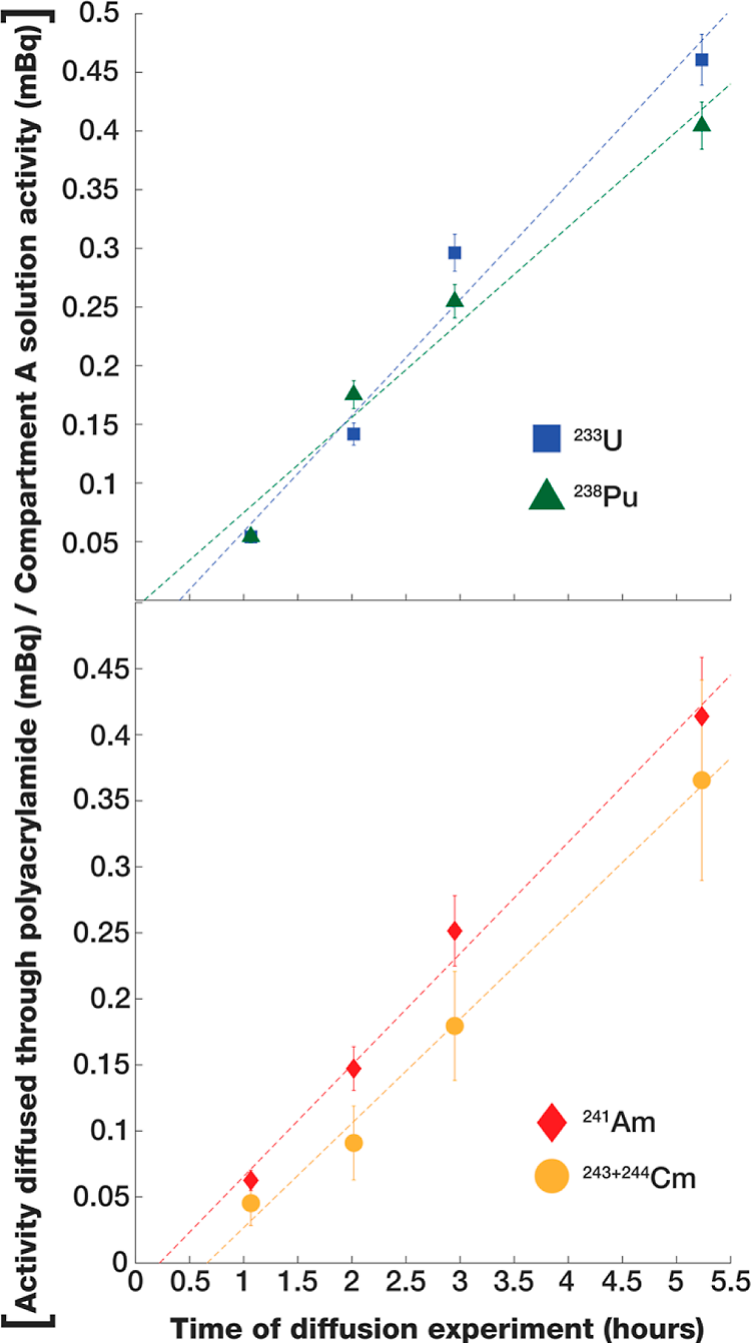
Diffusion of the actinides (in BASS) into compartment B of the diffusion cell vs time.

The linear temporal diffusion of the actinides through the polyacrylamide diffusive gel is demonstrated in Figure 1. The linear temporal uptake of the actinides by the fully assembled KMS-1 and IIP-Y^3+^ DGT samplers has previously been demonstrated.^1^ Additionally, the adsorbent functionalities of the KMS-1 and IIP-Y^3+^ resins used in the DGT resin gels have been shown to be effective at both lower and higher pH than the BASS solution.^9−13^ This also offers the reassurance that the KMS-1 resin will not break down and potentially liberate ions such as sulphates and chlorides into the SFP water.

### Considerations for Practical Implementation

We delivered  and isotopic report data to the nuclear facility with whom we collaborated for deployments of the DGT samplers in their SFP. This data is confidential, and we do not therefore present it in this work. In this section, we assess the deployments of the KMS-1 and IIP-Y^3+^ DGT configurations in the SFP as a proof of concept and offer considerations for their implementation.

The KMS-1 and IIP-Y^3+^ DGT configurations can be deployed either in small-surface-area (3.14 cm^2^) or large-surface-area (105 cm^2^) DGT sampler housings. Therefore, the deployment period of the samplers in SFP water is flexible, allowing for both short assays and also more detailed analyses of the SFP water. In our case, we deployed 105 cm^2^ surface-area DGT samplers in the SFP for a period of 24 h. A longer deployment period may be used to provide a more valid time-weighted average assessment of the actinides, especially if there are fluctuations in the temperature of the fuel pool water that may impact labile actinide concentrations. In any case, the average temperature of the SFP water throughout the deployment period must be known to correct the BASS-calibrated  presented in Table 1. We recommend that the sample holder is manufactured from pure stainless steel to avoid any reaction with the SFP water.

In the case that detailed data is desired rather than an assay of the SFP water concentrations, we recommend that four samplers are simultaneously deployed during each measure: two of each the KMS-1 and IIP-Y^3+^ configurations, containing polyacrylamide gel diffusive layers of different thickness (0.39 and 0.78 mm). Deriving  from the  calculated from the samplers with different thicknesses as described by Warnken et al.^14^ can indicate whether stagnant conditions in the SFP water have enlarged the  in front of the DGT device during deployment, allowing  and therefore  to be further fine-tuned as a function of the conditions in the specific SFP. We also note that different nuclear facilities may use boron enriched in ^11^B in order to reduce the concentration of boric acid required in the SFP. If the H_3_BO_3_ concentration is significantly different than that used in this work, further calibration of *D* in BASS at the relevant concentration using a diffusion cell may increase the accuracy of the calculated .

The use of AMS offers an ultrasensitive method to detect low-abundance isotopes and provide detailed isotopic reports, which are useful to assess the neutron flux of the reactor and the consequent burn-up of the nuclear fuel. The production of such isotopic ratios (e.g., ^240^Pu/^239^Pu and ^242–246^Cm isotope abundances) is not possible by using alpha spectrometry, which has energy interferences which do not allow the distinction of ^240^Pu from ^239^Pu, ^243^Cm from ^244^Cm, and ^245^Cm from ^246^Cm. We note that in the case of our measures, we were comfortably able to detect ^236^U (a low-abundance activation product isotope) in the gas ionization chamber of the TANDY AMS^6^ in the order of >200 000 total counts. However, the high-abundance ^238^U and ^235^U isotopes are measured concurrently by TANDY in a Faraday cup (FC) as an ion current.^6^ The sensitivity of the FC is many orders of magnitude lower than that of the gas ionization chamber. In our case, there was not enough ^238^U and ^235^U in the sample measurable in the FC. ^238^U and ^235^U would be comfortably analyzed by AMS in most other sample types which contain the same quantity of ^236^U, such as environmental samples which do not have an exceptionally high ^236^U/^238^U ratio as our samples of neutron-irradiated nuclear fuel. However, in this case, ^238^U and ^235^U in an aliquot of the U fraction may be measured by ICP–MS or α-spectrometry if this data is desired.

In a small aliquot of the resin-gel eluates that we measured by γ-spectrometry, we did not detect any significant activities of fission or activation products. This is beneficial for the handling transport of the samplers if laboratory analysis is not performed on-site. We note however that the KMS-1 resin has been shown to capture Sr^2+^ and Cs^+^,^12,13^ which we consider are also most likely the dominant Sr and Cs species under the physicochemical conditions within SFP water. Additionally, IIP-Y^3+^ may capture other 3+ radionuclide cations in the fuel pool water. This means that scope may exist to investigate the capabilities of the KMS-1 and IIP-Y^3+^ DGT configurations for the analysis of other fission and activation products in SFP, possibly given the measure of larger aliquots of the resin-gel eluates and with longer deployment times. The spike–unspiked methodology of measuring Am and Cm in the same fraction by AMS in this work allows for a rapid analysis of these actinides in the IIP-Y^3+^ resin-gel eluate. We consider that the potential limitation of a small presence of ^243^Am in the SFP water would not significantly affect the  for ^243^Cm calculated using this method. However, further work could explore implementing the separation of Am and Cm for their measure in separate fractions, as, for example, seems effective by oxidizing Am(III) to Am(V) using 0.01 M AgNO_3_.^15^

### Assessment of the Cm Chronometry Technique

The identification of Cm in the samplers and the calculated time of the retirement of a leaking fuel rod(s) from the neutron flux were blindly presented to colleagues at the nuclear facility where we deployed the samplers. It was confirmed to us that the fuel age that we calculated corresponded with a period when fuel rods were known to be leaking at the facility (PERS.COMM). This is the first application of this Cm chronometry technique with independent data. The correspondence of these ages therefore serves as a preliminary validation for the application of this technique. This method provides significant advantages over current nuclear fuel dating techniques, including that no tracer addition is required, and crucially, neither is the analysis of two separate elements which could chemically fractionate during analysis. This technique could therefore be a useful contribution to international nuclear security for the identification of rogue fuel fragments. This work also shows that this methodology can be employed to trace a leaking fuel rod in SFPs.

### Conclusions

We endorse the KMS-1 and IIP-Y^3+^ DGT configurations to provide determinations of U, Pu, Am, and Cm in SFP water based on our data, which demonstrates linear temporal diffusion of the actinides in simulated SFP water. The ability to measure a full range of actinide isotopes by using AMS provides isotopic reports from the DGT sampler eluates which are useful to assess the neutron flux of the reactor and date a leaking fuel rod.
